# A call for consensus in defining efficacy in clinical trials for opioid addiction: combined results from a systematic review and qualitative study in patients receiving pharmacological assisted therapy for opioid use disorder

**DOI:** 10.1186/s13063-019-3995-y

**Published:** 2020-01-06

**Authors:** Brittany B. Dennis, Nitika Sanger, Monica Bawor, Leen Naji, Carolyn Plater, Andrew Worster, Julia Woo, Anuja Bhalerao, Natasha Baptist-Mohseni, Alannah Hillmer, Danielle Rice, Kim Corace, Brian Hutton, Peter Tugwell, Lehana Thabane, Zainab Samaan

**Affiliations:** 1 McMaster University Internal Medicine Residency Program, Department of Medicine, Faculty of Health Sciences, McMaster University, Hamilton, Canada; 2 Department of Health Research Methods, Evidence, and Impact, McMaster University, Hamilton, Canada; 3 Department of Family Medicine Residency Program, Michael G. Degroote School of Medicine, McMaster University, Hamilton, Canada; 4 Department of Psychiatry and Behavioural Neurosciences, McMaster University, 1280 Main Street West, Hamilton, Ontario L8S 4L8 Canada; 5 Department of Medicine, Hamilton General Hospital, Hamilton, Canada; 6 University of Toronto Faculty of Medicine, University of Toronto, Toronto, Canada; 7 Faculty of Science, Department of Psychology, McGill University, Montreal, Canada; 8 Center for Practice Changing Research, Ottawa Hospital Research Institute, Ottawa, Canada; 9 Faculty of Medicine, University of Ottawa, Ottawa, ON Canada; 10 University of Ottawa Institute of Mental Health Research, Ottawa, ON Canada; 11 Clinical Epidemiology Program, Ottawa Hospital Research Institute, Ottawa, Ontario Canada; 12 School of Epidemiology and Public Health, University of Ottawa, Ottawa, Ontario Canada; 13 WHO Collaborating Centre for Knowledge Translation and Health Technology Assessment in Health Equity, Bruyère Research Institute, Ottawa, Canada; 14 Centre for Evaluation of Medicine, Hamilton, Canada; 15 System Linked Research Unit, Hamilton, Canada; 16 Population Genomics Program, Chanchlani Research Center, McMaster University, Hamilton, Canada

**Keywords:** Opioid addiction, Clinical trials, Efficacy, Methodology, Patient important outcomes, Treatment effectiveness

## Abstract

**Background:**

Given the complex nature of opioid addiction treatment and the rising number of available opioid substitution and antagonist therapies (OSAT), there is no ‘gold standard’ measure of treatment effectiveness, and each successive trial measures a different set of outcomes which reflect success in arbitrary or opportune terms. We sought to describe the variation in current outcomes employed across clinical trials for opioid addiction, as well as determine whether a discrepancy exists between the treatment targets that patients consider important and how treatment effectiveness is measured in the literature.

**Methods:**

We searched nine commonly used databases (e.g., EMBASE, MEDLINE) from inception to August 1, 2015. Outcomes used across trials were extracted and categorized according to previously established domains. To evaluate patient-reported goals of treatment, semi-structured interviews were conducted with 18 adults undergoing methadone treatment.

**Results:**

We identified 60 trials eligible for inclusion. Once outcomes were categorized into eight broad domains (e.g., abstinence/substance abuse), we identified 21 specific outcomes with furthermore 53 subdomains and 118 measurements. Continued opioid use and treatment retention were the most commonly reported measures (46%, *n* = 28). The majority of patients agreed that abstinence from opioids was a primary goal in their treatment, although they also stressed goals under-reported in clinical trials.

**Conclusions:**

There is inconsistency in the measures used to evaluate the effectiveness of OSATs. Individual and population level decision making is being guided by a standard of effect considered useful to researchers yet in direct conflict with what patients deem important.

**Trial registration:**

PROSPERO, CRD42013006507.

## Background

Information retrieved from the highest quality evidence—most often from randomized controlled trials (RCTs)—is used to inform health care decisions at individual and population levels. From the development of research questions to decisions regarding “significant” treatment targets, the research community exerts a strong influence on the generation of evidence. The end users of this evidence—whether this be physicians, policy makers, or patients—rely on the expert opinion of researchers to design studies and ultimately trust they select the appropriate outcomes to reflect treatment success. Despite best interests, the value of many pharmacological interventions is commonly evaluated on their observed effect across different biochemical and surrogate measurements [1]. Frequently these measurements neither reflect nor acknowledge the values and preferences of the populations they are meant to serve. Patient-important outcomes reflect the health concerns, fears regarding adverse drug reactions, treatment goals, and overall values of patient populations. These outcomes are often underrepresented in comparison to biologic measurements closely associated with the physiologic disease process [1]. For instance, the majority of trials within the diabetes literature include primary endpoints such as blood glucose level as an indicator of efficacy due to its direct relation to the pathophysiology of diabetes. Outcomes such as death, stroke, infection, pain function, or delayed wound healing have significant impact on patients’ lives, yet are often underreported [2]. Unfortunately, patient-important outcomes are often neglected in trials aimed to establish treatment benefit; this deficit is of substantial concern to the growing evidence base in opioid addiction, known formally as opioid use disorder (OUD) [3].

Given the complex nature of OUD treatment and the rising number of available pharmacological opioid substitution and antagonist therapies (OSAT), there is no “gold standard” measure of treatment effect and each successive trial measures a different set of treatment outcomes which reflect success in arbitrary or opportune terms [4–6]. Commonly included endpoints comprise attrition rates, illicit substance use, presence of medical and psychiatric comorbidity, social function as measured by current housing arrangements, collective neighborhood income, educational achievement, employment, and involvement in criminal activity [4–6]. The variation in the selection of outcomes as well as the marked range of definitions, instruments, and measurements of specific outcomes demand the need for further research to establish a summary of the current outcomes utilized in the literature, as well as determine which outcomes reflect patients’ values and preferences for the end goals of addiction treatment.

In the current study, we sought to outline the current outcomes employed in clinical trials for opioid addiction, as well as to determine whether a divide exists between the treatment targets patients consider important and those selected to evaluate efficacy in the literature.

## Methods

This study was completed in two phases. In the first phase of the study we completed a systematic review which aimed to describe outcomes used in the current literature to establish effectiveness of different OSATs. The second phase aimed to determine patient’s perspectives of successful addiction treatment with emphasis on the patient’s end-goals of therapy. Phase 1 of this study used the previously published protocol for a systematic review and network meta-analysis comparing OSAT interventions for OUD during which we also extracted the listing of outcomes reported within each study [7]. The literature search was completed in August 2015; this was not updated for the current study given that the emphasis is not on establishing a superior therapy for addiction, but rather to provide a summary of the outcome measures employed across clinical trials comprising the main body of evidence.

### Phase 1: systematic review to establish outcomes used in the current literature

#### Methodology

The collective body of evidence for OSAT trials was identified using results from a previous systematic review. A summary of the methods for this work are described in the published protocol [7]. The original systematic review utilized for this study was registered in the PROSPERO database (CRD42013006507) and adheres to the PRISMA guidelines [8].

Studies included in the previously published review were limited to trials evaluating pharmacological therapies for opioid addiction in general addiction populations; any studies in special populations, including prison, were excluded. No studies were eliminated based on outcome selection. All primary investigators listed on the NIH Clinical Trial Registry from eligible studies identified during the title screening were contacted for inquires regarding any publications resulting from their trials. The original review placed no constraints on language or date of publication. Animal studies and incomplete studies (pilot, preliminary reports) were excluded. Methodological quality assessment was conducted using the Cochrane Risk of Bias Tool for RCTs.

#### Summary of outcomes used across OSAT trials

The primary aim of the current study was to summarize all outcome domains and subdomains and their definitions and outcomes measurements/instruments used for each outcome in trials of OSAT for OUD. Data extraction forms were constructed and pilot tested for use in this review. We abstracted the sample size, mean age, eligibility criteria, intervention description, dose, approaches to missing data, outcome definition, outcome measurement, covariates included in regression models if adjusted analyses were performed, and the statistical association reported (e.g., odds ratio (OR), relative risk (RR)).

To provide an organized summary, we structured outcomes into broader categories according to the domains proposed by commonly used measurement scales evaluating addiction severity (i.e., the Addiction Severity Index (ASI) [9] and Maudsley Addiction Profile (MAP)) [10]. These tools evaluate treatment response using the broader domains of substance use behavior, physical and mental health, and social functioning [9, 10]. Both tools are practical and provide a global assessment of patients’ physical and social functioning. Our outcome domains included physical health, psychiatric health and symptoms, abstinence and substance use behavior, and personal and social functioning. Some studies used additional outcomes that did not conform to these domains; thus, we included global quality of life and addiction severity assessments (including global addiction severity, intervention adherence, acceptance of intervention, and resource utilization (e.g., hospital admission) as additional domains. This categorization of outcome domains and subdomains provides researchers and clinicians with an overview of the current outcomes used to assess patients’ responses to OSAT.

All outcomes used across trials included in this review were extracted and categorized according to the above described criteria.

### Phase 2: qualitative interviewing of patients on pharmacological treatment for OUD

#### Recruitment and interview methodology

Patients were recruited from two opioid addiction treatment centers in Ontario, Canada using purposive sampling. The research collaborative between the Population Genomics Program at McMaster University and the Canadian Addiction Treatment Centers (CATC) provided a framework for study recruitment, data collection, data analysis, and follow-up. Eligibility criteria included: patients ≥ 18 years, currently receiving an opioid substitution therapy including methadone maintenance treatment or buprenorphine, able to understand and speak English, and able to provide informed consent [11]. The Hamilton Integrated Research Ethics Board (HiREB) approved this study (HiREB study ID 0168). This study adheres to the STROBE guidelines [12].

Qualitative methods were used to establish patients’ perspectives of successful addiction treatment. Structured open-ended interviews were conducted to explore each patient’s end-goals of therapy. These interviews identified common themes with regard to addiction treatment goals. The interviews were transcribed and analyzed for themes, clarifications, and deeper understanding of the topics outlined above [11].

Convenience sampling was utilized between two addiction treatment clinics. Recruiting from two separate sites allowed for a broader patient demographic to be covered, as socioeconomic status and homelessness rates were known to differ between sites. Flyers advertising the study were posted at both clinical sites. All patients eligible for recruitment were also approached and informed about the study objectives by the clinic’s healthcare staff. Upon gaining informed consent, patients were given a demographic questionnaire and interviewed by two investigators using structured questions and open-ended questions. No one else was present at these interviews. All patients included in the study were given a $5 gift card at the end of the interview [11].

Interviews were conducted by an addiction specialist nurse, Carolyn Platter (BScNurs), and two female research coordinators, Julia Woo (BHSc) and Anuja Bhalerao (BHSc). These team members have performed hundreds of interviews in this population since working with the McMaster GENOA research collaborative. The interviewers were selected in efforts to minimize potential bias generated during data collection. These team members had no previous stake in the research question or design of this study. All interviewers underwent ethics and sensitivity training prior to meeting the patients, as per McMaster University Research Ethics Board Guidelines. Each team member has completed the Tri-Council Policy Statement course. The patients recruited into the study had not been previously interviewed by the team members and we are confident there was no relationship between participants and interviewers prior to the interview. Participants were briefed as to the goals of the study, particularly our aim of establishing whether current research accurately reflects what they wish to gain from treatment.

Interviews were completed using a structured piloted questioning tool with prompts; patients were approached allowing for open-ended answers. Each interview was audio recorded for later transcription. Each interviewer also made field notes, which were used to aid in later transcription. Each interview transcript was carefully investigated for insight into the major research question, “How would you measure success in methadone maintenance or buprenorphine treatment?” We also provided patients a list of commonly anticipated treatment goals and asked them to rank which aspect of recovery meant the most to their addiction treatment. Patients were allowed to rank up to four items. The list provided a summary of different potential goals across substance abuse, physical health, emotional stability, and personal functioning domains. A register of these goals in addition to the interview tool can be found in Additional file 1. The interviews lasted approximately 40 min and were conducted on site at the treatment facility between September 2015 and February 2016. Interviews were conducted until responses to the major research questions were saturated, having no new themes emerge [11]. Patients were not provided transcribed copies of their interview.

#### Analysis

Interviews were transcribed and evaluated for the common definitions of success in addiction treatment as well as aspects of recovery patients found important. Two primary interviewers (AB, JW) were responsible for coding the data, unaided by software. This process was later reviewed by all members of the team. These responses were coded according to the broader domains proposed by popular measurement scales evaluating addiction severity: the ASI [9] and MAP [10]. Additional domains not included in the MAP or ASI were also added. These domains included global quality of life and addiction severity assessments (including global addiction severity measure scores), intervention adherence, acceptance of intervention, and resource utilization (e.g., hospital admission) [11].

Due to the small size and limited power of our sample, no statistical tests were conducted in reference to significant differences between the participants at the two sites [11].

## Results

### Phase I: findings from the systematic review

**Fig. 1:**
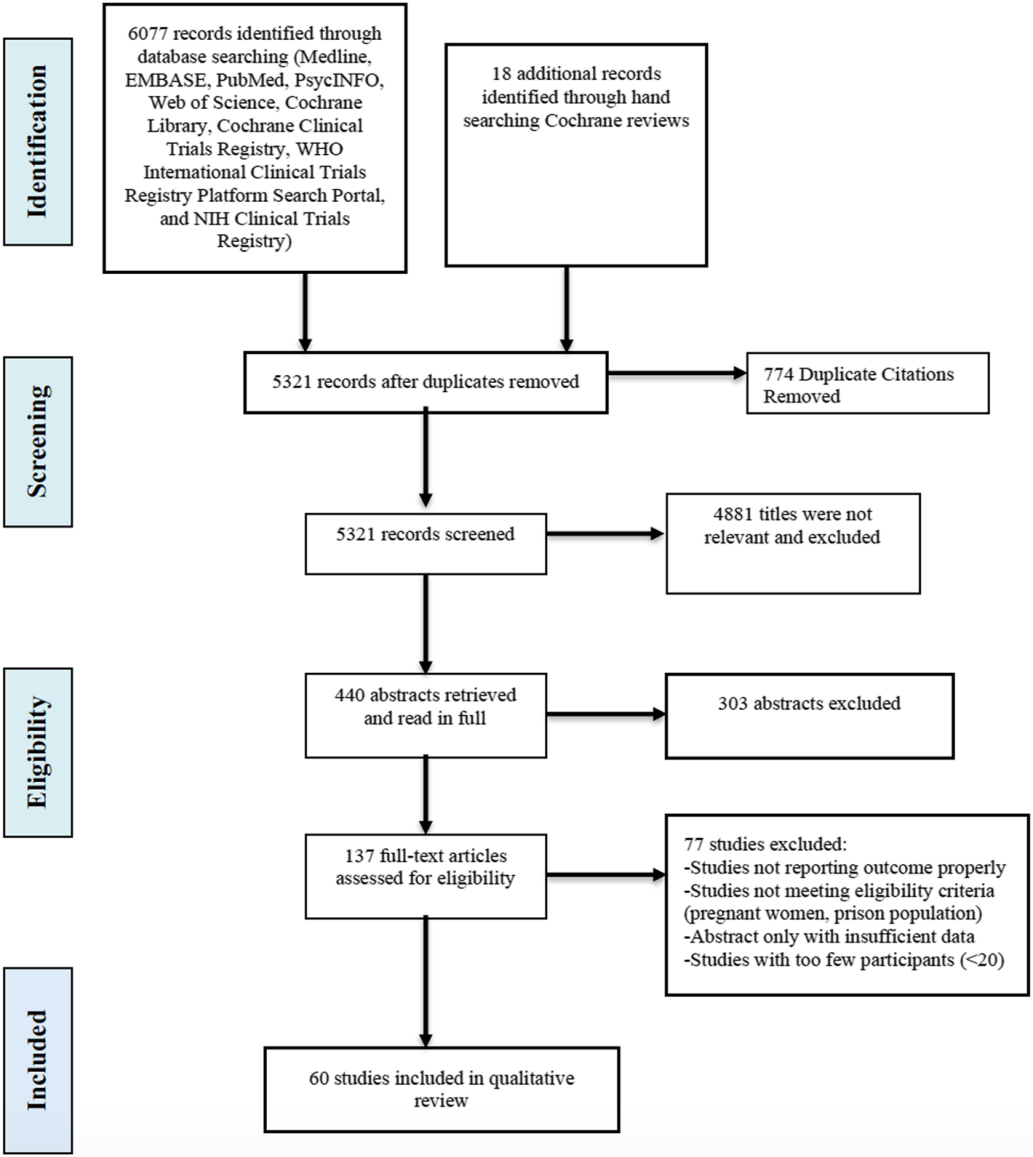
The preferred reporting items for systematic reviews and meta-analyses (PRISMA) flow diagram

An annotated flow diagram of the study selection process is presented in Fig. 1. We searched databases since inception to August 1, 2015 and identified 6077 articles. We identified 60 trials with a combined participant sample of 13,341 patients eligible for full text extraction [13–72]. A summary of the included trials is available in Additional file 1. Table 1 summarizes the outcome domains and subdomains used across trials included in this study; the outcomes are categorized into broad domains, outcome domains, subdomains, and the specific measurements. Within the eight broadest domains (abstinence and substance use behavior, physical health, psychiatric health and symptoms, personal and social functioning, resource utilization, intervention adherence, intervention acceptance, and global quality of life and addiction severity) are 21 more specific outcome domains (e.g., illicit opioid use, illicit non-opioid substance use), and across these outcomes there exist 53 separate definitions or measurements.

**Table 1:** Summary of all clinical and social outcomes used to establish effectiveness for trials in opioid addiction

Domains	Outcomes	Subdomains	Measurement of Outcome
Abstinence and substance use behavior	Illicit opioid use	Frequency of illicit opioid use (mean number of negative opioid urine screens or percentage of positive opioid screens, days of illicit use, assessed per treatment arm)	Urine toxicology screening
			A composite score from the Addiction Severity Index (European version)
			Self-report
			Hair sample toxicology screening
			Scores from Addiction Severity Index (American interview) domain assessing number of days of opiate use in last month
			Visual analog scale (daily heavy drug abuse was recorded as 10 and ‘drug free’ was recorded as 0)
			Weekly activity summary (WAS)
		‘Dirty rate’ measured using the number of opiate-positive urine screenings divided by the number of weeks of study participation	Urine toxicology screening
		Time to relapse measured using the number of days between baseline and occurrence of the first opiate-positive urine screening	Urine toxicology screening
		Failure to maintain abstinence	Urine toxicology screening
		Heroin use in preceding month at 3-, 6-, and 12-month interviews	Self-reported frequency of use measured using the Opiate Treatment Index
		Response to treatment measured as a reduction of regular use of street heroin, which was defined as 50% or more of negative specimens on urinalysis during weeks	Urine toxicology screening
		Percentage of patients in a drug free period, defined as time elapsed between the first day of naltrexone administration and the first evidence of opiate abuse (day on which positive urine test for opiate was obtained, or alternatively, the day on which the patient reported on opiate abuse)	Urine toxicology screening
		Abstinence from street heroin (zero use) in the past 30 days	Self-reported abstinence obtained by independent researchers in face-to-face interviews
		Assessment of near (< 2 opioid positive urine screens) and full abstinence (0 opioid positive urine screens)	Urine toxicology screening
		Percentage of participants per treatment arm who maintained 12 consecutive opioid-free urine screens	Urine toxicology screening
		Slip defined as occasional heroin use, less than three consecutive positive urine screens, and no symptoms of withdrawal	Self-report and urine toxicology screening
		Days to heroin relapse (three consecutive opiate-positive urine screens)	Urine toxicology screening
		Number of days a patient could remain abstinent measured by the longest duration of opiate negative urine screen	Urine toxicology screening
		Drug use history and routes of substance abuse	Risk Behaviour Survey
		The global severity of all aspects of their current drug problem	Self-report on a scale of 0 (no problem) to 100 (very severe)
		Opioid relapse defined as everyday heroin use, three consecutive positive urine tests, or reported symptoms of withdrawal	Self-report and urine toxicology screening
		Degree of opioid substance abuse	Global rating scale: rating of 2 marked an improvement in rehabilitation and substance use
	Non-opioid Substance Use	Frequency of poly-substance use (e.g., percentage/mean number of positive stimulants/benzodiazepines urine screens per treatment arm cocaine, benzodiazepines, illicit methadone)	Self-report
			Reported by family members or friends watching the participant
			Weekly activity summary (WAS)
			Visual analog scale (daily heavy drug abuse was recorded as 10 and ‘drug free’ was recorded as 0)
			Weekly Drug Use Questionnaire
			Urine toxicology screening
		Days of alcohol use per treatment arm	Self-report
		Severity of nicotine dependence	The Fagerström Test for Nicotine Dependence
		Alcohol consumption	Breathalyzer test
		The global severity of all aspects of their current drug problem	Measured on a scale of 0 (no problem) to 100 (very severe)
		Drug use history and routes of substance abuse	Risk Behaviour Survey
	Health risk behavior related to substance use	Injecting drug-use behavior	Self-report
		Reduction in HIV risk behaviors	AIDS risk inventory
			Opiate Treatment Index
			Risk Assessment Battery (RAB) scores
			Maudsley Addiction Profile
	Money spent or gained on illicit opioid consumption	Amount of money spent on illicit opioid consumption per month	Addiction Severity Index
		Amount of money gained from illicit opioid consumption per month	Addiction Severity Index
Physical health	Drug cravings	Craving for opioid substances	Subjective Opiate Withdrawal Scale, German version
			Visual Analog Scale for Heroin Craving
			Craving Visual Analogue Scale (CVAS; administered every week): a 10 cm line—with an end corresponding to 0 and the other to 100—was used to record the extent of subjective cravings for heroin, cocaine. and alcohol in the preceding week
			Tiffany Heroin Craving Questionnaire
	Overdose	Overdose of illicit or prescribed opioid and non-opioid substances requiring medical attention	Self-report
			Medical chart review
	Withdrawal Symptoms	Opioid physical withdrawal symptoms	The Withdrawal Symptoms Checklist
			Self-reported euphoric feelings
			The Addiction Severity Index
			Subjective Opiate Withdrawal Scale (German version: SOES)
			Self-report
			The Wang Scale
			Addiction Research Centre Inventory
	General physical health	General physical health and well-being, an assessment of current physical symptoms, physical functioning, physical role limitations, bodily pain, physical comorbidity, as well as medical history	Opioid Treatment Index
			Quality of Life scale (SF-12)
			Self reported health measured assessing symptoms, overdoses, and mortality
			Maudsley Addiction Profile
			Short Form 36-item Health Survey
		Physicians perception of disease severity and overall improvement compared to baseline	Clinical Global Impressions Scale, German Version
		Immune system functioning	Plasma concentrations of TNF-alpha, IL-2 beta, IL-1beta and CD14 lymphocyte
		Cardiac function assessed with corrected QT interval measurements	Electrocardiographic analysis
		Evaluation of patients meetings the categorical QTc prolongation thresholds across treatment groups (e.g., more than 470 ms for males and more than 490 ms for females)	Electrocardiographic analysis
Psychiatric health and symptoms	Psychiatric symptoms	Psychiatric assessment for depression, anxiety, and other psychiatric symptoms	Mental health symptoms measured using the SF-12
			Symptom checklist-90 (SCL-90)
			Short Form 36-item
			Self-rating Depression (SRD) questionnaire
			Minnesota Multifactorial Personality Inventory (MMPI)
			Symptom checklist (SCL-5)
			The Beck Depression Inventory
			State Trait Anxiety Inventory (STAI)
			Sensation Seeking Scale (SSS)
			Addiction Severity Index
			Maudsley Addiction Profile
			Scale of Anhedonia syndrome
			Self-reported assessments (somatization, depression, hostility, anxiety, paranoid ideation, interpersonal sensitivity)
	Psychological adjustment	Psychological and social adjustment	Addiction Severity Index (family and social relations scores)
			Opiate Treatment Index (social functioning scores)
			Clinical Global Impression as assessed by the Brief Psychiatric Rating Scale
Global quality of life and addiction severity assessments (outcomes of combined domains)	Composite addiction severity scores	Composite scores from addiction severity assessments that encompass patients physical, psychological, and social functioning, as well as their substance use behavior	Composite International Diagnostic Interview
			European Addiction Severity Index
			Addiction Severity Index
	Global quality of life	Quality of life assessment encompasses the evaluations of physical, social, physical, and psychological well-being	SCL-90-R subscales
			SCL-90-R global scores
			General Symptomatic Index
			Positive Symptom Total
			Positive Symptom Distress Index
			Lancashire Quality of Life Profile
			Visual analog scale (10 = very bad, 0 = very well) and with the temporal satisfaction with life scale (TSLS)
Personal and social functioning	Criminal behavior	Involvement in illegal activity	Self-reported days involved in illegal activities
			Self-reported time spent with people still abusing substances, selling drugs, engaging in illegal activity
			Lifestyle Changes Questionnaire (patients indicated whether they had engaged in any of nine activities to stop, reduce, or avoid cocaine/heroin use during the past week and whether they had committed crimes)
			Weekly Activity Summary (WAS 42)
	Employment and social involvement	Social stability assessed using current employment, volunteer, or social activities	Self-reported changes in vocational and social rehabilitation
			Self-reported consumption of meals, type of accommodation, and current employment activities
			Weekly Activity Summary (WAS 42)
			Behavioral observation where the research assistant recorded (yes/no) if patients had initiated new activities or increased the amount of time spent in any of three activity categories: (1) employment; (2) family/social; and (3) personal (spiritual, counseling or psychotherapy, physical fitness)
			Participation in non-study related addiction treatment programs (Narcotics Anonymous, etc.)
	Relationships	Evaluation of relationships and personal conflict with others	Personal and social functioning domain of the Maudsley Addiction Profile
			Social functioning measured using SF-36 health survey
			Personal and social function measured by self-reported time spent with people still abusing substances, selling drugs, engaging in illegal activity
	Personal stability	Evaluation of personal stability through assessment of housing and food consumption	Self-reported consumption of meals and type of accommodation
Resource utilization	Service utilization	Evaluation of how patients utilize available treatment and social services	Days patients were seen by counselors
			Total clinic attendance
Intervention adherence	Retention in treatment	Number of patients remaining on the allocated intervention at the end of follow-up	Adjudicated by the trial research staff
		Number of patients remaining on the allocated intervention and maintained a standard of opioid-free urine set by the study coordinators at the end of follow-up	Adjudicated by the trial research staff
		Time until patient withdraws from treatment	Adjudicated by the trial research staff
	Intervention compliance	Days patients attended clinic as an assessment of how well patient adheres to the treatment regime	Adjudicated by the trial research staff
			Treatment attendance, the number of days medicated divided by days in treatment
			Involvement of a significant other in treatment who was asked to supervise and report on compliance at each study visit, either in person or by telephone
		Assessment of medication adherence (evaluation of whether patient takes the medication prescribed)	Visual inspection of urine, inclusion of riboflavin 50 mg in the active and placebo naltrexone capsules with visual inspection for its presence using ultraviolet light at the long wave setting (444 nm) in a room with low ambient light
			Count of remaining capsules at each appointment
			Study patients were required to respond to a random medication recall once each 4 weeks to monitor and deter potential misuse of methadone
		Involvement in services provided by treatment centers	Assessment of the counseling visits, which was based on the length (minutes) and number of contacts the patient had with either individual or group treatments
	Successful medication induction	At least one dose of medication by the sixth day of the study	Assessed by clinical research staff
Intervention acceptance	Intervention preference	Assessment of final drug of choice (at end of cross-over trial participants could chose which therapy to remain on)	Self-report
		Medication preferences (includes a proxy assessment of dosing adequacy)	The Helping Alliance Questionnaire II (HAq-II; patient version), a 19-question self-administered instrument that measures the quality of therapeutic alliance between patients and therapists from the point of view of the patients
			The Client Satisfaction Questionnaire (CSQ), a self-administered questionnaire that assesses overall satisfaction with treatment
			Measured using a visual analogue questionnaire of drug properties which required them to “rate each drug on six different factors: is the drug holding (suppressing withdrawal); how much buzz do you get from the drug; do you experience side effects; do the side effects bother you; do you like the drug; and do you feel more normal?”

Of the 60 trials eligible for inclusion in this review, retention in treatment was the most commonly measured and reported outcome. Of the 28 studies reporting retention in treatment as their primary outcome, 16 different interventions were evaluated. The second most commonly reported outcome was illicit opioid use, which took 17 definitions and a further eight variations in measurement. The wide-ranging definitions for illicit opioid use included 1) the frequency of use in the form of the mean number or days of use or the percentage of positive urine screens, 2) the mean time patients remain abstinent on therapy or time until the first positive opioid urine screen is observed, 3) the number of participants per treatment arm who fulfill a predefined criteria for “success” or “failure” according to their opioid use consumption patterns, and 4) the global severity of opioid use as scored from a validated tool. Further variations arose based on the measurement of opioid use, which included urine toxicology screening with directly observed or non-observed sampling, toxicology screening with hair samples, validated addiction severity measurement tools, as well as weekly activity summaries or self-report.

General physical health outcomes comprised the largest differences in both conceptualization and measurement. Physicians’ perception of disease, cardiac function, immune system function, pain severity, and the presence of physical comorbidity were among the commonly measured aspects of general physical health.

### Phase II: qualitative interviewing of patients on pharmacological treatment for opioid addiction

A total of 18 individuals from two treatment centers participated in this study. Sixteen of the participants were currently undergoing MMT at the time of recruitment and two participants were receiving buprenorphine but had received MMT at least one year prior. The mean age of the participants was 36.11 (standard deviation = 10.01) years with majority female (67%) and of Caucasian ethnicity (89%). Participants in one site had a higher mean income ($48,750 vs $35, 000) and were more likely to be employed (63% vs 40%) compared to the second site’s participants, which is expected as the second site was selected purposefully to be economically different. All participants were interviewed in a single session and no repeated sessions were necessitated during the course of this study.

#### Qualitative interview

The majority of participants (61.1%) identified their main goal of methadone treatment as being abstinence from drugs. This goal was clearly indicated by patients, including statements like: “Just being completely off of drugs. To never touch drugs again.” Close to a third of these individuals had a more specific goal of being off of methadone completely (38%). One participant stated specifically (as seen in the following direct quote) that even though they are sober, their ultimate goal is to be “clean” from all opioids.

“*When someone tells me I’m not sober because I’m on methadone. I tell them I may not be clean because I’m putting this medication in my body but I am sober. I want to be clean. To me, I’m sober right now, I have been sober for two and a half years. I haven’t touched the drugs for two and a half years. At the end of it, I want to be off the methadone completely but I want to be able to taper down till I no longer need it anymore and I want to look back and say that was just a phase in my life. I took the necessary steps to make myself better and I accomplished that. And all the things that I accomplished being on methadone too. So yeah, I just want to get off of it completely, eventually”*

Others did not desire to be off methadone and specified methadone was helping them. One participant’s main goal was for pain control and not to be off methadone, as it helped them function and be able to move. When asked if they were hoping to get completely off methadone they responded saying, “I don’t know if I ever will. I see my doses being reduced but until my health problems are resolved, I have absolutely no problem being on it if it has to be for the rest of my life.”

Other goals of methadone treatment that were not as common included being able to get back to their usual lives and able to maintain it, to not be sick, and to manage addictions not only related to drugs but in other domains of their life. Participant’s verbatim responses are summarized in Table 2. The percentages presented above reflect an assessment of patient responses presented in Table 2.

**Table 2:** Verbatim answers to qualitative interview to understand goals of therapy

Participant	Verbal answer
1	Remain abstinent from drugs
2	I don’t want to use drugs
3	Not use street drugs
4	Get off opioids completely
5	Maintain my job
6	Just get my life back; I’m still an addict and I don’t want that to sneak back on me
7	To not be sick anymore
8	Being completely off drugs. To never touch drugs again
9	Being able to control my addiction. Just living a life without having to take medication every day
10	Not to use drugs
11	Being independent from methadone and drugs
12	Pain control
13	To get off methadone and never look back at any opioids
14	Managing my addictive personality, whether it is a drug addiction or not
15	Get clean; not going back on opioid and not go back on Suboxone
16	Become drug free
17	Get off methadone; be done with this all
18	Get off it (methadone) completely

#### Response to predetermined treatment goals

**Fig. 2:**
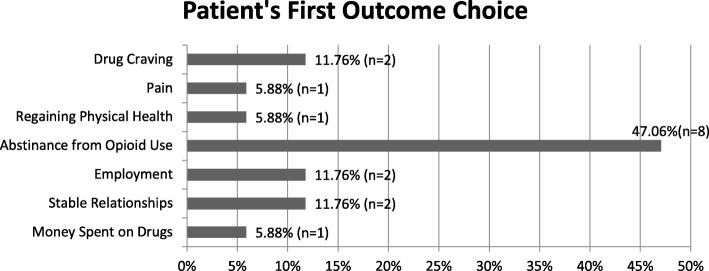
First ranked treatment goals among patients receiving OSAT. Patients ranking of treatment goals from a “pre-determined” list provided during the qualitative interview. Patients were asked to rank which aspect of recovery was most important to their addiction treatment goals. Patients were allowed to rank up to four items. The figure illustrates the first ranked items

Seventeen out of the 18 participants completed the section indicating which aspect of recovery meant the most to their addiction treatment. Please refer to Fig. 2 for a graphical summary of patients’ first ranked treatment goals. This graphical summary was generated using the individual patient data reported in Table 3, whereby the frequency of participants ranked goals of care was calculated and subsequently presented as a percentage.

**Table 3:** Patients’ responses to predetermined treatment goals

Participant	Outcome 1	Outcome 2	Outcome 3	Outcome 4
1	Money spent on drugs	Overdose	Injecting	NA
2	Stable relationships	Coping	NA	NA
3	Employment	Housing	Depression	
4	Stable relationships	Money spent on drugs	Sexual function	Money spent on drugs
5	Employment	Stable relationships	Housing	NA
6	Abstinence from opioid use	Employment	NA	NA
7	Regaining physical health	Abstinence from opioid use	NA	NA
8	Abstinence from opioid us	Regaining physical health	Coping	NA
9	Missing data			
10	Abstinence from opioid use	Depression	Coping	NA
11	Abstinence from opioid use	Drug craving	Money spent on drugs	Regaining physical health
12	Pain	Employment	NA	NA
13	Abstinence from opioid use	Money spent on drugs	Drug craving	Stable relationships
14	Drug craving	Stable relationship	Money spent on drugs	NA
15	Drug craving	Pain		
16	Abstinence from opioid use	Pain	Stable relationships	Drug craving
17	Abstinence from opioid use	Money spent on drugs	Depression	Anxiety
18	Abstinence from opioid use	Drug craving	Stable relationships	NA

*NA* not available

Abstinence from opioid use was the most commonly selected outcome overall followed by stability of relationships, reduced money spent on drugs, reduced drug craving, employment, regaining physical health, pain control, coping, reduced depression, stable housing, improved sexual function, decreased risk of overdose, reduced injecting, and reduced anxiety overall across all participants’ four outcome choices. The most commonly selected primary outcome for participants was abstinence from opioid use, with 47% (8) of participants selecting it as their first choice; 16.6% (3) chose money spent on drugs as their second most important outcome. Participant’s outcomes are summarized in Table 3.

## Discussion

Findings from this study outline the current outcomes employed in clinical trials for opioid addiction, and also provide a unique insight into the treatment goals patients consider important when receiving pharmacological therapies for OUD.

Results from the secondary review of outcome measures employed in OUD trials highlights a major lack of consensus in our evidence base when determining appropriate end-points for establishing treatment effectiveness for OSATs. A substantial number of outcomes as well as variations in the definitions and measurements of the same outcomes were reported across trials. Despite the overwhelming collection of outcomes employed by trialists, substance use—specifically opioid—and treatment retention remain the most consistently reported. Trialists seldom explored pharmacological effect on personal and social functioning outcomes such as criminal behavior, employment, relationships, and personal stability end-points, including type of accommodation (20%, *n* = 12).

The most commonly employed outcomes used to establish effectiveness were in stark contrast to the goals for treatment patients described in the qualitative interviews performed for the second phase of our study. While the majority of patients agreed that abstinence and reduction in opioid use was a primary goal in their treatment, they also stressed goals for therapy comprising employment, improved relationship stability, reduction in the money spent on drugs, as well as improvements in physical and psychiatric symptoms such as pain, depression, and anxiety. Regrettably, these outcomes were rarely reported or of primary focus in the clinical trials.When assessing the comparative effectiveness of all interventions among patients receiving OSATs, retention in treatment was the most consistently measured and reported outcome across trials (46%, *n* = 28). In direct contrast to staying on treatment, our interviews with patients demonstrate an eagerness to complete therapy and get off the methadone treatment regime as a recurrent theme.

Outcome selection bears serious implications for the interpretation of the results as well as our ability to extrapolate such findings in a wider clinical context. These methodological shortcomings highlight the need for new assessment strategies for opioid addiction treatment options, where future efforts should consider targeting the objective assessment of treatment effectiveness employing long-term follow-up using administrative data-linkage for trial participants to evaluate hard long-term outcomes such as incidence of hepatitis, HIV, cardiovascular abnormalities, and mortality. Among the trials included in this review, three evaluated the impact of interventions on mortality [41, 60] or cardiac function [44].

Trials evaluating OSATs suffer from poor methodological quality [73]. A combination of small sample size, poor design, highly stringent eligibility criteria, effect estimates with tremendous imprecision, short-follow up time, missing data, and a major lack of consensus over patient-important outcomes has led to an accumulation of a large yet very weak body of evidence. Whether it be illicit opioid use or risky behavior, the large number of definitions and measurements used to assess the same attribute suggest the need for more consensus in the field and understanding of what treatment outcomes are most important to addiction patients.

The evidence generated for this review was gathered from our previous work which aimed to determine the most effective pharmacotherapy for opioid use disorder [7]. An important finding from our original included the lack of standardization in outcome selection, in addition to the overall absence of discourse on patient-important outcomes in opioid use disorder. We felt strongly that this topic required a thorough discussion in a stand-alone paper and would be further complemented by the addition of qualitative interviews establishing patient values and preferences. We acknowledge the limitations posed by not updating our search strategy for the current study, particularly the lack of representation of studies conducted since the onset of the opioid crisis. However, our emphasis remains not establishing a superior therapy for addiction, which would require the most up-to-date assessment of all evidence, but rather to provide a summary of the outcome measures employed across clinical trials comprising the main body of evidence, which is largely captured in our current review, and likely would remain unchanged.

Efforts to map the health values and preferences of these 18 participants across all outcomes identified in the systematic review would have provided unique perspective to our current evaluation of the evidence. We hesitated to perform this analysis in light of the small sample size and absence of full representation of the outcome domains and subdomains identified from our review in the interview tool. Thus, any effort to draw conclusions regarding the representation of patient values in trial outcome selection could be explained by our lack of representation of the full list of trial outcomes in the interview tool.

Involvement of participants from our qualitative study phase in order to obtain a group consensus of the most valued goals of care would have been an instrumental addition to our evaluation of current OUD outcomes. Unfortunately, we did not hold ethics approval for that type of focus group work. It is clear a core outcomes set is needed in the field of OUD, which will require a larger, more representative study of all stakeholders. We maintain the key objectives of this work were to generate a discourse for patient-important outcomes in the OUD literature, and ultimately to provide the foundation for future researchers to explore this question in a larger representative sample.

## Conclusions

In agreement with current guidelines, our study demonstrates there is limited consistency in the outcomes used to evaluate the effectiveness of OSATs [74, 75]. More concerning, our treatment recommendations and clinical decisions are being guided by a standard of effect considered useful to researchers yet in direct conflict with what patients deem important. This is a substantial limitation in the literature. Without the identification of a measurable treatment outcome that has an impact on and significance to patients, services, and the population as a whole, all the investment in trials will result in inadequate and inconsistent “efficacy” with limited, if any, external validity. We demonstrate here the need for an established set of OSAT outcomes guided by all stakeholders to inform clinicians of the true efficacy of these therapies and guide trialists to ensure our future understanding of these treatments accurately reflects the priorities of our patient population.

## Supplementary information

**Additional file 1. **Interview tool: the interview tool used in qualitative interviews. **Table S1.** Summary of included trials: table summarizing important information from all trials included in this systematic review, including the journal, number of participants, and Cochrane risk of bias score.

## Data Availability

The datasets used and/or analyzed during the current study are available from the corresponding author on reasonable request.

## Abbreviations

GENOA: Genetics of opioid addiction
HiREB: Hamilton Integrated Research Ethics Board
OSATs: Opioid substitution and antagonist therapies
OUD: Opioid use disorder
PRISMA: Preferred reporting items for systematic reviews and meta-analyses
RCT: Randomized controlled trial
