# Supplementary material for: A call for consensus in defining efficacy in clinical trials for opioid addiction: combined results from a systematic review and qualitative study in patients receiving pharmacological assisted therapy for opioid use disorder
Source: Trials. 2020 Jan 6;21:30. doi: 10.1186/s13063-019-3995-y (PMC6945391; doi:10.1186/s13063-019-3995-y)
Supplement: Supplementary file 1 — Additional file 1. Interview tool: the interview tool used in qualitative interviews. Table S1. Summary of included trials: table summarizing important information from all trials included in this systematic review, including the journal, number of participants, and Cochrane risk of bias score. [file 13063_2019_3995_MOESM1_ESM.pdf]

Qualitative Study of the Effects of Stigma on Methadone Users

Project Number: 15-168

Interview Format

Introduction: The purpose of this study is to gain a deeper understanding of the sources and effects of stigma faced by methadone maintenance therapy (MMT) users. We will be using structured, open-ended questions to explore this topic. These questions will allow us to gain deeper understanding of stigma surrounding drug addiction and methadone, and identify potential ways of improving MMT users' recovery and wellbeing.

This interview will be approximately an hour in length and open-ended. You do not have to answer any questions you feel uncomfortable about. You can choose to pause or stop the interview at any time. Do not hesitate to ask us for clarification if you feel unsure about any of our questions.

As well, the interview will be audio recorded to ensure that we are collecting all the relevant information. The interview recordings will be kept in password-protected files to which only the researchers have access. All files will remain in a secure McMaster server, accessible through McMaster's Virtual Private Network only.

1. How would you define recovery in your own words? What do you think a "successful methadone treatment outcome" looks like? What would your measure of success be?
    a. PROMPT: Would you say it is abstinence you're looking for? Employment? Mending relationships?
    b. PROMPT: Is the stopping of illegal opioid use the only thing you consider when improving addictive behaviour?
    c. PROMPT: Are there other aspects of your addictive behaviour that you wish to change?
    d. PROMPT: What types of goals do you set at the beginning of your treatment regime? Do they include improving your mental and physical health?

2. Is there anything else that you would like to add? Do you have any questions or concerns?

Supplementary Web Appendix

Qualitative Study of Patient Important Outcomes in Addictions Research

Treatment Outcomes

Which of the following aspects of recovery mean the most to you? How would you measure success in methadone maintenance treatment?

- Employment
- Housing
- Stable relationships
- Regaining physical health
- Sexual function
- Criminal activity
- Money spent on drugs
- Emergency room visits
- Hepatitis and other STDs
- Abstinence from opioid use
- Overdose
- Drug craving
- Injecting
- Opioid use
- Cocaine use
- Pain
- Coping
- Depression
- Anxiety

Supplementary Web Appendix Table 1: Summary of Included Trials

Author Last Name	Journal	Number of Participants	Cochrane Risk of Bias Score
Ahmadi, 2003[1]	Irish Journal of Medical Science	204	11
Van Den Brink, 2003[2]	BMJ	549	18
Comer, 2006[3]	Arch Gen Psychiatry	60	15
Anglin, 2007[4]	Addiction	315	10
Fischer, 1999[5]	Addiction	60	11
Fudala, 2003[6]	New England Journal of Medicine	326	16
Giacomuzzi, 2006[7]	Substance Use and Misuse	120	14
Haasen, 2007[8]	The British Journal of Psychiatry	1015	15
Johnson, 1992[9]	JAMA	162	17
Krupitsky, 2012[10]	Arch Gen Psychiatry	306	18
Ling, 1996[11]	Arch Gen Psychiatry	225	15
Schottenfeld, 2008[12]	The Lancet	126	18
Johnson, 1995[13]	Drug and Alcohol Dependence	150	12
Ahmadi, 2004[14]	Journal of Substance Abuse Treatment	513	11
Ahmadi, 2002[15]	Drug and Alcohol Dependence	330	11
Yancovitz, 1991[16]	Am J of Pub Health	301	11
Sees, 2000[17]	JAMA	179	16
Strain, 1994[18]	The American Journal of Psychiatry	164	14
Soyka, 2008[19]	International Journal of Neuropsychopharmacology	140	10
March, 2006[20]	Journal of Substance Abuse Treatment	62	13
Eder, 2005[21]	Addiction	64	17
Eissenberg, 1997[22]	JAMA	180	18
Jaffe, 1972[23]	JAMA	34	15
Kamien, 2008[24]	Heroin Addiction and Related Clinical Problems	268	18
King, 2006[25]	Journal of Substance Abuse Treatment	92	15
Oviedo-Joekes, 2009[26]	The New England Journal of Medicine	226	14
Oviedo-Joekes, 2010[27]	Drug and Alcohol Review	54	17

Potter, 2013[28]	Journal of Studies on Alcohol and Drugs	731	14
Robertson, 2006[29]	Addiction	235	12
Saxon, 2013[30]	Drug and Alcohol Dependence	1269	15
Schwartz, 2011[31]	Journal of Substance Abuse Treatment	230	16
Shwartz, 2006[32]	Archives of General Psychiatry	319	14
Strain, 1996[33]	Journal of Clinical Psychopharmacology	86	17
Wolstein, 2009[34]	Pharmacopsychiatry	84	12
Ling, 1976[35]	Arch Gen Psychiatry	142	13
White, 2002[36]	Drug and Alcohol Dependence	62	13
Kosten, 1993[37]	J Nervous Mental Disease	125	18
Wedam, 2007[38]	Arch Intern Med	102	15
Woody, 2008[39]	JAMA	152	14
Kakko, 2003[40]	Lancet	40	17
Krook, 2002[41]	Addiction	106	16
Ling, 1998[42]	Addiction	736	18
Lintzeris, 2004[43]	American Journal on Addictions	139	10
Mattick, 2003[44]	Addiction	405	17
Neri, 2005[45]	Psychopharmacology	62	16
Shufman, 1994[46]	Biological Psychiatry	32	16
Pani, 2000[47]	Drug and Alcohol Dependence	72	14
Schottenfeld, 1998[48]	The Journal of Nervous and Mental Disease	116	15
Preston, 2000[49]	Arch Gen Psychiatry	285	18
Schottenfeld, 2005[50]	American Journal of Psychiatry	162	16
Strain, 1993[51]	Annals of Internal Medicine	247	15
Hartnoll, 1980[52]	Arch Gen Psychiatry	96	17
Strang, 2010[53]	Lancet	127	18
Zaks, 1972[54]	JAMA	20	10
Strain, 1999[55]	JAMA	192	17
Petitijean, 2001[56]	Drug and Alcohol Dependence	58	17
Guo, 2001[57]	Hong Kong Journal of Psychiatry	302	15
Krupitsky, 2004[58]	Journal of Substance Abuse Treatment	52	15
San, 1991[59]	British Journal of Addiction	50	15
Krupitsky, 2006[60]	Journal of Substance Abuse Treatment	280	18

* Cochrane risk of bias scores are summed from individual ranking among multiple subdomains, giving a total score out of 18. Higher scores indicate increasing risk of bias.

Study References from Appendix Table 1

1. Ahmadi J, Ahmadi K. Controlled trial of maintenance treatment of intravenous buprenorphine dependence. *Irish journal of medical science.* Oct-Dec 2003;172(4):171-173.
2. van den Brink W, Hendriks VM, Blanken P, Koeter MW, van Zwieten BJ, van Ree JM. Medical prescription of heroin to treatment resistant heroin addicts: two randomised controlled trials. *Bmj.* Aug 9 2003;327(7410):310.
3. Comer SD, Sullivan MA, Yu E, et al. Injectable, sustained-release naltrexone for the treatment of opioid dependence: a randomized, placebo-controlled trial. *Arch Gen Psychiatry.* Feb 2006;63(2):210-218.
4. Anglin MD, Conner BT, Annon J, Longshore D. Levo-alpha-acetylmethadol (LAAM) versus methadone maintenance: 1-year treatment retention, outcomes and status. *Addiction.* Sep 2007;102(9):1432-1442.
5. Fischer G, Gombas W, Eder H, et al. Buprenorphine versus methadone maintenance for the treatment of opioid dependence. *Addiction (Abingdon, England).* 1999;94(9):1337-1347. http://onlinelibrary.wiley.com/o/cochrane/clcentral/articles/437/CN-00265437/frame.html.
6. Fudala PJ, Bridge TP, Herbert S, et al. Office-based treatment of opiate addiction with a sublingual-tablet formulation of buprenorphine and naloxone. *N Engl J Med.* Sep 4 2003;349(10):949-958.
7. Giacomuzzi SM, Ertl M, Kemmler G, Riemer Y, Vigl A. Sublingual buprenorphine and methadone maintenance treatment: a three-year follow-up of quality of life assessment. *ScientificWorldJournal.* May 24 2005;5:452-468.
8. Haasen C, Verthein U, Degkwitz P, Berger J, Krausz M, Naber D. Heroin-assisted treatment for opioid dependence: randomised controlled trial. *Br J Psychiatry.* Jul 2007;191:55-62.
9. Johnson RE, Jaffe JH, Fudala PJ. A controlled trial of buprenorphine treatment for opioid dependence. *Jama.* May 27 1992;267(20):2750-2755.
10. Krupitsky E, Zvartau E, Blokhina E, et al. Randomized trial of long-acting sustained-release naltrexone implant vs oral naltrexone or placebo for preventing relapse to opioid dependence. *Archives of general psychiatry.* 2012;69(9):973-981. http://onlinelibrary.wiley.com/o/cochrane/clcentral/articles/942/CN-00841942/frame.html.
11. Ling W, Wesson DR, Charuvastra C, Klett CJ. A controlled trial comparing buprenorphine and methadone maintenance in opioid dependence. *Archives of general psychiatry.* 1996;53(5):401-407. http://onlinelibrary.wiley.com/o/cochrane/clcentral/articles/562/CN-00124562/frame.html.
12. Schottenfeld RS, Chawarski MC, Mazlan M. Maintenance treatment with buprenorphine and naltrexone for heroin dependence in Malaysia: a randomised, double-blind, placebo-controlled trial. *Lancet.* Jun 28 2008;371(9631):2192-2200.
13. Johnson RE, Eissenberg T, Stitzer ML, Strain EC, Liebson IA, Bigelow GE. A placebo controlled clinical trial of buprenorphine as a treatment for opioid dependence. *Drug Alcohol Depend.* Nov 1995;40(1):17-25.

14. Ahmadi J FH, Moosavinasab M, Babaee M, Firoozabadi A, Mohagheghzadeh M., et al. Treatment of heroin dependence. *German Journal of Psychiatry.* 2004;7(2):1-5.
15. Ahmadi J. A controlled trial of buprenorphine treatment for opium dependence: the first experience from Iran. *Drug and alcohol dependence.* 2002;66(2):111-114. http://onlinelibrary.wiley.com/o/cochrane/clcentral/articles/791/CN-00384791/frame.html.
16. Yancovitz SR, Des Jarlais DC, Peyser NP, et al. A randomized trial of an interim methadone maintenance clinic. *Am J Public Health.* Sep 1991;81(9):1185-1191.
17. Sees KL, Delucchi KL, Masson C, et al. Methadone maintenance vs 180-day psychosocially enriched detoxification for treatment of opioid dependence: a randomized controlled trial. *JAMA : the journal of the American Medical Association.* 2000;283(10):1303-1310. http://onlinelibrary.wiley.com/o/cochrane/clcentral/articles/101/CN-00276101/frame.html.
18. Strain EC, Stitzer ML, Liebson IA, Bigelow GE. Comparison of buprenorphine and methadone in the treatment of opioid dependence. *Am J Psychiatry.* Jul 1994;151(7):1025-1030.
19. Soyka M, Zingg C, Koller G, Kuefner H. Retention rate and substance use in methadone and buprenorphine maintenance therapy and predictors of outcome: results from a randomized study. *Int J Neuropsychopharmacol.* Aug 2008;11(5):641-653.
20. March JC, Oviedo-Joekes E, Perea-Milla E, Carrasco F. Controlled trial of prescribed heroin in the treatment of opioid addiction. *Journal of substance abuse treatment.* 2006;31(2):203-211. http://onlinelibrary.wiley.com/o/cochrane/clcentral/articles/751/CN-00571751/frame.html.
21. Eder H, Jagsch R, Kraigher D, Primorac A, Ebner N, Fischer G. Comparative study of the effectiveness of slow-release morphine and methadone for opioid maintenance therapy. *Addiction (Abingdon, England).* 2005;100(8):1101-1109. http://onlinelibrary.wiley.com/o/cochrane/clcentral/articles/083/CN-00529083/frame.html.
22. Eissenberg T, Bigelow GE, Strain EC, et al. Dose-related efficacy of levomethadyl acetate for treatment of opioid dependence. A randomized clinical trial. *JAMA : the journal of the American Medical Association.* 1997;277(24):1945-1951. http://onlinelibrary.wiley.com/o/cochrane/clcentral/articles/955/CN-00140955/frame.html.
23. Jaffe JH, Senay EC, Schuster CR, Renault PR, Smith B, DiMenza S. Methadyl acetate vs methadone. A double-blind study in heroin users. *JAMA : the journal of the American Medical Association.* 1972;222(4):437-442. http://onlinelibrary.wiley.com/o/cochrane/clcentral/articles/696/CN-00007696/frame.html.
24. Kamien JB, Branstetter SA, Amass L. Buprenorphine-naloxone versus methadone maintenance therapy: A randomised double-blind trial with opioid-dependent patients. *Heroin Addiction and Related Clinical Problems.* 2008;10(4):5-18. http://onlinelibrary.wiley.com/o/cochrane/clcentral/articles/368/CN-00754368/frame.html.
25. King VL, Kidorf MS, Stoller KB, Schwartz R, Kolodner K, Brooner RK. A 12-month controlled trial of methadone medical maintenance integrated into an adaptive treatment model. *J Subst Abuse Treat.* Dec 2006;31(4):385-393.
26. Oviedo-Joekes E, Brissette S, Marsh DC, et al. Diacetylmorphine versus methadone for the treatment of opioid addiction. *N Engl J Med.* Aug 20 2009;361(8):777-786.
27. Oviedo-Joekes E, March JC, Romero M, Perea-Milla E. The Andalusian trial on heroin-assisted treatment: a 2 year follow-up. *Drug Alcohol Rev.* Jan 2010;29(1):75-80.
28. Potter JS, Marino EN, Hillhouse MP, et al. Buprenorphine/naloxone and methadone maintenance treatment outcomes for opioid analgesic, heroin, and combined users: findings from starting treatment with agonist replacement therapies (START). *J Stud Alcohol Drugs.* Jul 2013;74(4):605-613.
29. Robertson JR, Raab GM, Bruce M, McKenzie JS, Storkey HR, Salter A. Addressing the efficacy of dihydrocodeine versus methadone as an alternative maintenance treatment for opiate dependence: A randomized controlled trial. *Addiction.* Dec 2006;101(12):1752-1759.
30. Saxon AJ, Ling W, Hillhouse M, et al. Buprenorphine/Naloxone and methadone effects on laboratory indices of liver health: a randomized trial. *Drug Alcohol Depend.* Feb 1 2013;128(1-2):71-76.

31. Schwartz RP, Kelly SM, O'Grady KE, Gandhi D, Jaffe JH. Interim methadone treatment compared to standard methadone treatment: 4-month findings. *J Subst Abuse Treat.* Jul 2011;41(1):21-29.
32. Schwartz RP, Highfield DA, Jaffe JH, et al. A randomized controlled trial of interim methadone maintenance. *Arch Gen Psychiatry.* Jan 2006;63(1):102-109.
33. Strain EC, Stitzer ML, Liebson IA, Bigelow GE. Buprenorphine versus methadone in the treatment of opioid dependence: self-reports, urinalysis, and addiction severity index. *Journal of clinical psychopharmacology.* 1996;16(1):58-67. http://onlinelibrary.wiley.com/o/cochrane/clcentral/articles/824/CN-00130824/frame.html.
34. Wolstein J, Gastpar M, Finkbeiner T, et al. A randomized, open-label trial comparing methadone and Levo-Alpha-Acetylmethadol (LAAM) in maintenance treatment of opioid addiction. *Pharmacopsychiatry.* 2009;42(1):1-8. http://onlinelibrary.wiley.com/o/cochrane/clcentral/articles/527/CN-00681527/frame.html.
35. Ling W, Charuvastra C, Kaim SC, Klett CJ. Methadyl acetate and methadone as maintenance treatments for heroin addicts. A veterans administration cooperative study. *Arch Gen Psychiatry.* Jun 1976;33(6):709-720.
36. White JM, Danz C, Kneebone J, La Vincente SF, Newcombe DA, Ali RL. Relationship between LAAM-methadone preference and treatment outcomes. *Drug Alcohol Depend.* May 1 2002;66(3):295-301.
37. Kosten TR, Schottenfeld R, Ziedonis D, Falcioni J. Buprenorphine versus methadone maintenance for opioid dependence. *The Journal of nervous and mental disease.* Jun 1993;181(6):358-364.
38. Wedam EF, Bigelow GE, Johnson RE, Nuzzo PA, Haigney MC. QT-interval effects of methadone, levomethadyl, and buprenorphine in a randomized trial. *Arch Intern Med.* Dec 10 2007;167(22):2469-2475.
39. Woody GE, Poole SA, Subramaniam G, et al. Extended vs short-term buprenorphine-naloxone for treatment of opioid-addicted youth: a randomized trial. *JAMA : the journal of the American Medical Association.* 2008;300(17):2003-2011. http://onlinelibrary.wiley.com/o/cochrane/clcentral/articles/502/CN-00651502/frame.html.
40. Kakko J, Svanborg KD, Kreek MJ, Heilig M. 1-year retention and social function after buprenorphine-assisted relapse prevention treatment for heroin dependence in Sweden: a randomised, placebo-controlled trial. *Lancet.* Feb 22 2003;361(9358):662-668.
41. Krook AL, Brors O, Dahlberg J, et al. A placebo-controlled study of high dose buprenorphine in opiate dependents waiting for medication-assisted rehabilitation in Oslo, Norway. *Addiction.* May 2002;97(5):533-542.
42. Ling W, Charuvastra C, Collins JF, et al. Buprenorphine maintenance treatment of opiate dependence: a multicenter, randomized clinical trial. *Addiction.* Apr 1998;93(4):475-486.
43. Lintzeris N, Ritter A, Panjari M, Clark N, Kutin J, Bammer G. Implementing buprenorphine treatment in community settings in Australia: experiences from the Buprenorphine Implementation Trial. *Am J Addict.* 2004;13 Suppl 1:S29-41.
44. Mattick RP, Ali R, White JM, O'Brien S, Wolk S, Danz C. Buprenorphine versus methadone maintenance therapy: a randomized double-blind trial with 405 opioid-dependent patients. *Addiction.* Apr 2003;98(4):441-452.
45. Neri S, Bruno CM, Pulvirenti D, et al. Randomized clinical trial to compare the effects of methadone and buprenorphine on the immune system in drug abusers. *Psychopharmacology (Berl).* May 2005;179(3):700-704.
46. Shufman EN, Porat S, Witztum E, Gandacu D, Bar-Hamburger R, Ginath Y. The efficacy of naltrexone in preventing reabuse of heroin after detoxification. *Biol Psychiatry.* Jun 15 1994;35(12):935-945.
47. Pani PP, Maremmani I, Pirastu R, Tagliamonte A, Gessa GL. Buprenorphine: a controlled clinical trial in the treatment of opioid dependence. *Drug Alcohol Depend.* Jul 1 2000;60(1):39-50.
48. Schottenfeld RS, Pakes JR, Kosten TR. Prognostic factors in Buprenorphine- versus methadone-maintained patients. *The Journal of nervous and mental disease.* 1998;186(1):35-43. http://onlinelibrary.wiley.com/o/cochrane/clcentral/articles/359/CN-00147359/frame.html.

49. Preston KL, Umbricht A, Epstein DH. Methadone dose increase and abstinence reinforcement for treatment of continued heroin use during methadone maintenance. *Arch Gen Psychiatry.* Apr 2000;57(4):395-404.
50. Schottenfeld RS, Chawarski MC, Pakes JR, Pantalon MV, Carroll KM, Kosten TR. Methadone versus buprenorphine with contingency management or performance feedback for cocaine and opioid dependence. *Am J Psychiatry.* Feb 2005;162(2):340-349.
51. Strain EC, Stitzer ML, Liebson IA, Bigelow GE. Dose-response effects of methadone in the treatment of opioid dependence. *Ann Intern Med.* Jul 1 1993;119(1):23-27.
52. Hartnoll RL, Mitcheson MC, Battersby A, et al. Evaluation of heroin maintenance in controlled trial. *Arch Gen Psychiatry.* Aug 1980;37(8):877-884.
53. Strang J, Metrebian N, Lintzeris N, et al. Supervised injectable heroin or injectable methadone versus optimised oral methadone as treatment for chronic heroin addicts in England after persistent failure in orthodox treatment (RIOTT): a randomised trial. *Lancet.* May 29 2010;375(9729):1885-1895.
54. Zaks A, Fink M, Freedman AM. Levomethadyl in maintenance treatment of opiate dependence. *JAMA : the journal of the American Medical Association.* 1972;220(6):811-813. http://onlinelibrary.wiley.com/o/cochrane/clcentral/articles/053/CN-00007053/frame.html.
55. Strain EC, Bigelow GE, Liebson IA, Stitzer ML. Moderate- vs high-dose methadone in the treatment of opioid dependence: a randomized trial. *Jama.* Mar 17 1999;281(11):1000-1005.
56. Petitjean S, Stohler R, Deglon JJ, et al. Double-blind randomized trial of buprenorphine and methadone in opiate dependence. *Drug Alcohol Depend.* Mar 1 2001;62(1):97-104.
57. Guo S. Efficacy of naltrexone Hydrochloride for preventing relapse among opiate dependent patients after detoxification. *Hong Kong Journal of Psychiatry* 2001;11(4):2-8.
58. Krupitsky EM, Zvartau EE, Masalov DV, et al. Naltrexone for heroin dependence treatment in St. Petersburg, Russia. *J Subst Abuse Treat.* Jun 2004;26(4):285-294.
59. San L, Pomarol G, Peri JM, Olle JM, Cami J. Follow-up after a six-month maintenance period on naltrexone versus placebo in heroin addicts. *British journal of addiction.* Aug 1991;86(8):983-990.
60. Krupitsky EM, Zvartau EE, Masalov DV, et al. Naltrexone with or without fluoxetine for preventing relapse to heroin addiction in St. Petersburg, Russia. *J Subst Abuse Treat.* Dec 2006;31(4):319-328.
